# Is Thymoglobulin or Rituximab the Cause of This Serum Sickness? A Case Report of Serum Sickness Dilemma and Literature Review

**DOI:** 10.1155/2012/234515

**Published:** 2012-09-27

**Authors:** Amarpreet Sandhu, Antonia Harford, Pooja Singh, Eduardo Alas

**Affiliations:** Department of Nephrology, The University of New Mexico, Albuquerque, NM 87131, USA

## Abstract

Serum sickness is an immune-complex-mediated systemic illness that can occur after treatment with monoclonal or polyclonal antibodies such as Rituxan (Rituximab) or antithymocyte globulin (Thymoglobulin), respectively. Since Rituximab is now being used as an adjuvant treatment for acute humoral rejection and its prevalence to cause serum sickness is comparable to Thymoglobulin-associated serum sickness (20% versus 27%), it should be considered a potential cause of serum sickness after rejection treatment. In kidney transplant patients, there are no case reports where patient received both Thymoglobulin and Rituximab before developing serum sickness. We are reporting a patient who developed serum sickness after receiving Thymoglobulin and Rituximab that led us to consider Rituximab as one of the potential causes in this patient's serum sickness. Since diagnosis of serum sickness is clinical, and Rituximab use has expanded into treatment of glomerulonephritis and acute humoral rejection, it should be considered as a potential offender of serum sickness in these patient populations. There are not any evidence-based guidelines or published clinical trials to help guide therapy for antibody-induced serum sickness; however, we successfully treated our case with three doses of Methylprednisone 500 mg intravenously. Further studies are needed to evaluate Rituximab-associated serum sickness in nephrology population to find effective treatment options.

## 1. Background

Rituxan (Rituximab), a partially humanized murine anti-CD20 monoclonal antibody developed to treat B cell lymphoma, has been used widely for autoimmune diseases. Now it is being used as adjuvant treatment of acute humoral rejection and many glomerulonephritis diseases. Rabbit-antithymocyte globulins (Thymoglobulin), polyclonal antibodies, are now used widely as induction immunosuppression in solid organ transplantation and treatment of acute cellular rejection of the allograft. Rituximab and Thymoglobulin can be used together to treat humoral and acute cellular rejection, and both can cause serum sickness.

Serum sickness, type III hypersensitivity, is an immune-complex-mediated reaction that results from generation of human immunoglobulins to circulating exogenous antigens. These immune complexes deposit into parenchymal tissues which then activate the complement cascade and ultimately result in systemic symptoms [1]. The frequency of serum sickness depends on the type of antigen exposure, and it approximately ranges from 0.007% with amoxicillin to 86% with horse antithymocyte globulin [2, 3]. In kidney transplant recipients, who receive Thymoglobulin, the incidence of serum sickness can be up to 27%, whereas the incidence is 20% in patients who receive Rituximab for treatment of autoimmune diseases [4, 5].

To the best of our knowledge, in kidney transplant patients, there are no case reports of serum sickness associated with Rituximab alone or with combined use of Thymoglobulin and Rituximab. However this case presentation led us to consider Rituximab as a potential or concomitant cause of serum sickness in this patient. We report a case of a patient who developed serum sickness and acute tubular necrosis after administration of Thymoglobulin and Rituximab to treat his acute cellular and humoral rejection.

Even though an abundance of literature is available about risk factors, presentation, and pathogenesis of serum sickness, no evidence-based guidelines or clinical trials exist to guide standard treatment for poly/monoclonal antibodies-induced serum sickness. We are presenting a case report of a serum sickness dilemma that was successfully treated with three doses of Methylprednisone 500 mg intravenously. Additionally, we will review the published literature of serum sickness treatment in this paper.

## 2. Case Report

A 51-year-old-male with history of deceased donor renal transplant, who 2 weeks prior to presentation was treated for acute cellular and humoral rejection, presented with fever, polyarthralgia, and swelling. His past renal history included end-stage renal disease from unclear etiology, a living-related renal transplant that he rejected within a week, a deceased donor renal transplant a year later, and hypertension. His other medical issues included hyperlipidemia, gout, and hypothyroidism. His treatment two weeks prior for his cellular and humoral rejection consisted of Methylprednisone 500 mg intravenous (IV) × 3 doses, plasmapheresis × 4 treatments, intravenous immunoglobulin (IVIG) 1 gram per kilogram per day × 2 doses, Rituximab 375 mg/m^2^ × 1 dose, and Thymoglobulin 1.5 mg/kg × 5 doses.

On presentation, he reported arthralgias which started in the right knee and were subsequently followed by arthralgias in the left shoulder and left wrist. He had a fever of 101.1°F (38.4°C), his left wrist was swollen on exam, and his right knee was tender with an effusion. The rest of the vitals and physical exam were unremarkable. Laboratory studies showed white cell count (WBC) of 7.9 × 10 E^3^, hemoglobin 9.7 g/dl, hematocrit 29%, platelets 157 × 10 E^3^, sodium 136 meq/l, potassium 4.2 meq/l, chloride 105 meq/l, carbon dioxide 21 meq/l, blood urea nitrogen (BUN) 41 mg/dl, serum creatinine (Scr) 1.8 mg/dl (baseline 1.7 mg/dl), total protein 6.7 g/dl, albumin 2.2 g/dl, AST 27 Unit/L, ALT 71 Unit/L, creatinine kinase 119 Unit/L, and uric acid 5 mg/dl. His wrist X-rays did not show any abnormalities, whereas his knee X-rays indicated a mild joint effusion of the right knee. Joint aspiration of the right knee showed total nucleated cells of 830 with neutrophils 62%, lymphocytes 0%, monocytes 38%, and no crystals were seen by polarized microscopy.

Given his hemodynamic stability, he was not started on empiric antibiotics; however, extensive workup was sent to rule out bacterial and viral infections. All of the evaluated viral serologies were negative and included Epstein-Barr-Virus (EBV), Cytomegalovirus (CMV), BK Virus (BKV), and Parvovirus B19 (Table 4). Rheumatologic workup showed low complement C3 and C4, and elevated erythrocyte sedimentation rate (ESR), C-reactive protein (CRP), haptoglobin, and lactate dehydrogenase (LDH) (Table 2).

In light of his recent treatment with Thymoglobulin and Rituximab, presenting symptoms, and negative infectious workup (Table 3), a presumptive diagnosis of serum sickness was made. His serum sickness was thought most likely secondary to Thymoglobulin but Rituximab was also high on differential, as delineated further in this paper. Heterologous antibodies were noted to be positive against rabbit IgG and IgM, low positive against horse IgG, and negative for mouse IgG (Table 4). Therapeutic plasma exchange (TPE) and corticosteroids are treatment options for Thymoglobulin-associated serum sickness; however, serum sickness from Rituximab has only been shown to be treated with corticosteroids. Given uncertain cause of his serum sickness, we started our patient on high dose Methylprednisone 500 milligrams IV per day for three days. He had complete resolution of polyarthralgia and swelling after the third dose of Methylprednisone.

During his hospitalization and treatment for his serum sickness, he developed acute kidney injury (AKI). A day after his third dose of Methylprednisone, his Scr peaked at 2.8 mg/dl from an initial baseline of 1.7 mg /dl (Table 2). His urinalysis was negative for infection, and urine microscopy showed granular and muddy brown casts consistent with acute tubular necrosis (ATN). Thus, his dose of furosemide was decreased to 20 milligrams from 40 milligram by mouth twice a day, and he was encouraged to drink more fluids. His renal function began improving on day no. 2 after-treatment with Methylprednisone, and Scr retuned back to his baseline of 1.7 mg/dl on day no. 6 after-treatment with Methylprednisone. 

## 3. Discussion

The term serum sickness was first coined in 1905 by von Pirquet and Schick in their book “Die Serumkrankheit” meaning serum sickness. They described it as illness that occurs after administration of horse serum given as antitoxin to treat diphtheria and scarlet fever [17]. Serum sickness results from generation of human immunoglobulins to circulating exogenous antigens and results in systemic symptoms. It commonly presents with fever, malaise, rash, polyarthralgia, and polyarthritis, one to two weeks after exposure to the offending agent. However, it can also present with less common symptoms such as headache, visual defects, facial or peripheral edema, splenomegaly, regional lymphadenopathy, and peripheral neuropathy [1].

The diagnosis of serum sickness can be suspected based on presentation, risk factors, and laboratory data. The risk factors include prior exposure, dose, duration, and nature of the heterologous protein. Few classic and rare laboratory findings include neutropenia or leukocytosis, mild thrombocytopenia, eosinophilia, elevated acute phase reactants such as ESR and CRP, low complement levels C3 and C4, and elevated circulating immune complexes such as C1q. Most of these laboratory findings were seen in our patient (Table 2). Other nonspecific laboratory findings include anti-heterologous antibodies against rabbit immunoglobulin IgG and IgM or human anti-chimeric antibody to Rituximab. According to one study [9], a history of raising (10.5%, *P* = 0.04) or ingesting (6.3%, *P* = 0.04) rabbits is associated with increased risk of serum sickness after receiving Thymoglobulin.

The diagnosis of serum sickness is clinical, and it can present with a variety of signs and symptoms, which may be concerning for an infectious process. Therefore, initiation of treatment is usually delayed until an infectious process is ruled out. Interim, most patients receive empiric treatment with antibiotics. Our patient presented with fever and symptoms concerning for infection, but given his hemodynamic stability and presumed serum sickness diagnosis, he was not started on antibiotics.

In solid organ transplant patients, many case reports of serum sickness after rabbit antithymocyte globulin use have been published but none after Rituximab use. There are approximately 20 case reports of Rituximab-associated serum sickness resulting after treatment of autoimmune diseases. Since Rituximab is now being used for treatment of glomerulonephritis and acute humoral rejection, and its prevalence to cause serum sickness is comparable to Thymoglobulin-associated serum sickness (20% versus 27%), it should be considered a potential cause of serum sickness in this patient population. Although most of the reported cases of Rituximab-associated serum sickness occur after more than or equal to 2 doses, there are case reports of serum sickness even with single dose of 375 mg/m^2^. Single dose of Rituximab is used in acute humoral rejection treatment whereas multiple doses are used in glomerulonephritis treatment [1] (Table 1). Our patient received one dose of Rituximab 375 mg per m^2^ and five doses of Thymoglobulin 1.5 mg per kg.

In the diagnosis of serum sickness, anti-heterologous and anti-chromeric antibodies can be tested in special laboratories that take weeks to result. Most patients are started on treatment without sending these tests or before the results are available. Anti-heterologous antibodies to rabbit IgG (Table 4) and rabbit exposure history were present in our patient, linking his serum sickness to his thymoglobulin treatment rather than Rituximab. However, sensitivity and specificity of this linkage are undetermined, and Rituximab still could be a cause of his serum sickness. According to one study [18], anti-rabbit antibodies were present in 8% of the kidney recipients, even before transplantation.

Common features of renal involvement in serum sickness include oliguria, proteinuria in about 50% of the patients, transient mild hematuria, and an elevated serum creatinine which can be twice the baseline creatinine. Acute tubular necrosis and glomerulonephritis can also occur due to a high renal blood flow and filtration effect. Immune complexes deposit in the subendothelial and mesangial matrix and activate the complement system which in turn leads to glomerulonephritis [19]. 

Since evidence-based guidelines or controlled trials to guide standard treatment for antibody-induced serum sickness are lacking, treatment choice can be difficult. The treatment of choice proved to be more difficult in our patient because he had received both Thymoglobulin and Rituximab two weeks prior to his serum sickness. Therapeutic plasma exchange (TPE) and corticosteroids are used as treatment options for serum sickness from Thymoglobulin, whereas serum sickness from Rituximab has only been shown to be treated with corticosteroids (Table 1). To date, there is no consensus on treatment modality for antibody-mediated serum sickness. Some studies report high dose steroids followed by a steroid taper as the preferred method [1]. Other studies used TPE as first line treatment or in steroid-resistant patients [9, 20]. We have outlined the different therapeutic options used for serum sickness thought to be caused by either Thymoglobulin or Rituximab in the literature (Table 1).

The use of high dose corticosteroids can cause side effects, such as psychosis, osteoporosis, adrenal insufficiency, and increased risk of infections. TPE as the first line treatment can cause risks of procedure complications and infections associated with central venous catheter placement especially in immunocompromised patients. In light of lacking data on TPE for Rituximab-related serum sickness, we chose high dose corticosteroids for our patient and successfully treated him with complete resolution of his symptoms and improvement of serum complement (Table 2).

In conclusion, serum sickness is an immune-complex-mediated systemic illness that can occur after treatment with monoclonal or polyclonal antibodies such as Rituximab or Thymoglobulin, respectively. Since Rituximab is now being used as an adjuvant treatment for acute humoral rejection and its prevalence to cause serum sickness is comparable to Thymoglobulin-associated serum sickness, it should be considered a potential or concomitant cause of serum sickness developed after rejection treatment. Currently, there are no evidence-based guidelines or clinical trials to guide treatment for antibody-induced serum sickness. It is even more difficult to determine the best course of treatment in patients who receive more than one offending agent. We successfully treated our case of serum sickness with a three-day course of high dose intravenous steroids, and it can be considered for serum sickness in patients who receive both Thymoglobulin and Rituximab for their rejection treatment. Since Rituximab is gaining its popularity as humoral rejection and glomerulonephritis treatment, further studies and case reports are needed to evaluate Rituximab-associated serum sickness to find optimal treatment options.

## Figures and Tables

**Table 1:** Monoclonal and polyclonal antibody-associated serum sickness and acute kidney injury case reports and treatment modalities.

Author	Patient Characteristics	Antibodies Used (ATG, ATGAM, or Rituximab)	Indication of Antibodies	Treatment Used	Cause of AKI and Methods	Specific treatment for AKI if Any
Snow et al. [6]	48-year-old female with CRT	Rabbit-ATG	Induction	Prednisone 60 mg PO/day for 2 days	Unknown	Conservative

Pham et al. [7]	Female with LRT	Rabbit-ATG	Acute Cellular Rejection	TPE × 2 treatments	Unknown	Conservative

	32-year-old male with CRT	Rabbit-ATG	Induction or Acute Cellular Rejection	1 mg/kg/day oral Prednisone or 2 mg/kg/day for 2 days Followed by TPE × 1-2 treatments	Unknown	Conservative
	60-year-old male with CRT	ATGAM	Induction or Acute Cellular Rejection	As Above	Unknown	Conservative
Tanriover et al. [4]	31-year-old male with CRT	Rabbit-ATG	Induction or Acute Cellular Rejection	As Above	N/A	N/A
	41-year-old male with LT	Rabbit-ATG	Induction or Acute Cellular Rejection	As Above	Unknown	Conservative
	39-year-old Female with CRT	Rabbit-ATG	Induction or Acute Cellular Rejection	As Above	N/A	N/A

Da Silva et al. [8]	10-year-old female with aplastic anemia	Rabbit-ATG	Aplastic Anemia	30 mg/kg/day of Methylprednisone for 3 days followed by prednisone taper	Unknown	Peritoneal Dialysis for 14 days

Boothpur et al. [9]	24-year-old female with LURT	Rabbit-ATG	Induction	TPE and Methylprednisone 500 mg IV for 2 doses	N/A	N/A
	20-year-old female with LURT	Rabbit-ATG	Induction	TPE × 3 treatments	N/A	N/A
	37-year-old male LURT	Rabbit-ATG	Induction	TPE × 2 treatments	N/A	N/A
	51-year-old male with LRT	Rabbit-ATG	Induction	Methylprednisone 500 mg IV × unknown doses followed by prednisone taper	N/A	N/A

Andresdottir et al. [10]	51-year-old male with kidney transplant	Horse-ATG	Induction	Unknown	Endocapillary Glomerulonephritis on Kidney biopsy	Conservative

Lundquist et al. [11]	45-year-old female with Liver Transplant	Rabbit-ATG	Induction	Methylprednisone 250 mg (3 mg/kg/day) × 3 doses	Unknown	Conservative

Disperati et al. [12]	52-year-old female with follicular lymphoma	Rituximab	Stage IV Follicular Lymphoma	IV Methylprednisone of unknown dose	N/A	N/A

DeMonaco and Jacobs [13]	47-year-old female with follicular lymphoma	Rituximab	Follicular Lymphoma	High dose corticosteroids followed by 10-day prednisone taper that failed. Then unknown doses of prednisone and indomethacin	N/A	N/A

D'Arcy and Mannik [14]	45-year-old male with refractory autoimmune polyneuropathy	Rituximab	Refractory Autoimmune Polyneuropathy	Pulse corticosteroids of unknown doses	N/A	N/A

Herishanu [15]	48-year-old female with refractory immune thrombocytopenia	Rituximab	Refractory Immune Thrombocytopenia	Methylprednisone 500 mg IV × 2 days	N/A	N/A

Hellerstedt and Ahmed [16]	23-year-old female with Lupus	Rituximab	Autoimmune Thrombocytopenia	Steroid bolus of unknown doses	N/A	N/A

Todd and Helfgott [1]	68-year-old male with stage 2A mantle cell lymphoma	Rituximab	Treatment for Mantle Cell Lymphoma	Intra-articular Methylprednisone 80 mg and Oral prednisone 20 mg daily followed by Prednisone taper for 4 weeks	N/A	N/A

Sandhu et al.	51-year-old male with CRT	Rabbit-ATG and Rituximab	Acute Cellular Rejection	SoluMedrol 500 mg IV × 3 days	Acute Tubular Necrosis findings on Urine Microscopy	Conservative

*ATG: antithymocyte globulin; ATGAM: antithymocyte globulin or lymphocyte immune globulin; TPE: therapeutic plasma exchange; CRT: cadaveric renal transplant; LRT: living-related transplant; LURT: living-unrelated transplant; IV: intravenous.

**Table 2:** Some of the serum sickness-specific laboratory findings before and after the treatment.

Test and Normal Range	At Presentation	After treatment	3 weeks Later
ESR (0–15 mm/hour)	105	NC	84
CRP (<0.3 mg/dL)	14.3	NC	<0.3
C3 (87–247 mg/dL)	74	NC	138
C4 (10–37 mg/dL)	7	NC	45
LDH (82–234 Unit/L)	244	NC	NC
Haptoglobulin (30–200 mg/dL)	422	NC	NC
BUN (3–21 mg/dL)	41	69	32
SCr (0.8–1.3 mg/dL)	1.8	2.8	1.7
Upr/Cr	6.1	2.4	2.0

NC: not checked; ESR: erythrocyte sedimentation rate; CRP: C-reactive protein; C3/C4: complements; LDH: lactate dehydrogenase; BUN: blood urea nitrogen; Scr: serum creatinine; Upr/Cr: urine protein to creatinine ratio.

**Table 3:** Serological and specific laboratory findings.

Other Serologies and Tests (Normal Values)	Patient
TSH (0.358–3.7 UIU/mL)	1.190
Rheumatoid Factor (<15 IU/mL)	<10
Antinuclear Antibody Screen (Negative)	Negative
Anti-Ds DNA (0–99 Unit/mL)	4
Anti-neutrophil Cytoplasm Antibodies (Neg)	Negative
Anti-Smith antibodies (0–99 Unit/mL)	7
Anti-Ribonuclear Protein Antibodies (0–99 Unit/mL)	28
Anti-Sclerosis Antibodies (0–99 Unit/mL)	14
SS-A(RO) Precipitin (0–99 Unit/mL)	8
SS-B(LA) Precipitin (0–99 Unit/mL)	9
CCP Antibody IGG (0–5 Unit/mL)	0.5
Epstein-Barr Virus IGG Antibody (Negative)	Positive
Epstein-Barr Virus IGM Antibody (Negative)	Negative
Parvovirus by PCR (Negative)	Negative
BK Virus by PCR (Negative)	Negative
Direct Coombs Test (Negative)	Negative
HIV 1/2 Antibody (Negative)	Negative
Creatine Kinase (35–232 Unit/L)	119
Uric Acid (3.5–7.2 mg/dL)	5
Total Protein (6.4–8.2 g/dL)	6.7
Albumin (3.4–5.8 g/dL)	2.2
AST (10–37 Unit/L)	27
ALT (12–78 Unit/L)	71
Alkaline Phosphatase (50–136 Unit/L)	59
Total Bilirubin (0.2–1 mg/dL)	0.3

TSH: thyroid stimulating hormone; CCP antibody IGG: cyclic citrullinated peptide antibody IGG; PCR: polymerase chain reaction; AST: aspartate aminotransferase; ALT: alanine aminotransferase.

**Table 4:** Quantitative heterologous antibody profile.

Heterologous Antibodies	Normal Ranges	Patient
Rabbit IgG	Negative = <1 : 100	1 : 3200
	Low Positive = 1 : 100–1 : 400	
	Mid Positive = 1 : 800–1 : 1600	
	High Positive = >1 : 1600	

Horse IgG	Negative = <1 : 100	1 : 100
	Low Positive = 1 : 100–1 : 400	
	Mid Positive = 1 : 800–1 : 1600	
	High Positive = >1 : 1600	

Mouse IgG	Negative = <1 : 100	Negative
	Low Positive = 1 : 100–1 : 400	
	Mid Positive = 1 : 800–1 : 1600	
	High Positive = >1 : 1600	

## Abbreviations

IVIG: Intravenous immunoglobulin
Thymoglobulin: Rabbit antithymocyte globulins
IV: Intravenous
WBC: White blood cell count
BUN: Blood urea nitrogen
Scr: Serum creatinine
EBV: Epstein-Barrvirus
CMV: Cytomegalovirus
BKV: BK virus
ESR: Erythrocyte sedimentation rate
CRP: C-reactive protein
LDH: Lactate dehydrogenase
TPE: Therapeutic plasma exchange
AKI: Acute kidney injury
ATN: Acute tubular necrosis.
